# Sister Study Launched Nationwide

**DOI:** 10.1289/ehp.112-a812

**Published:** 2004-10

**Authors:** Jennifer Medlin

Breast cancer is the second most commonly diagnosed form of cancer among U.S. women, according to the National Cancer Institute (NCI), and the second leading cause of cancer deaths in this group. For the year 2003 alone, the NCI estimated more than 212,000 new diagnoses and more than 40,000 deaths from breast cancer. A woman’s risk increases with her age: breast cancer is the most common cause of cancer death after age 65, and nearly half of all breast cancers are found in this age group—a figure that is likely to increase significantly as baby boomers age into the next decade. Plus, black women have the highest death rates from breast cancer. To address these concerns, the NIEHS-sponsored Sister Study plans to explore on a nationwide basis, beginning in October 2004, how genetic and environmental influences may work together to cause breast cancer.

The study has been in development since 2001 and under way in pilot form for the past year. A total of 50,000 female volunteers aged 35–74 whose sisters have been diagnosed with breast cancer will be recruited and receive health evaluations over a period of 10 or more years. First-degree relatives, including sisters, have about twice the risk as the average woman of developing breast cancer.

Past studies have focused on pesticides, solvents, and electromagnetic fields as possible contributors to breast cancer, but have failed to find consistent links to the disease. Study authors Dale Sandler, chief of the NIEHS Epidemiology Branch, and Clarice Weinberg, chief of the Biostatistics Branch, believe this study—unlike past studies—will be able to effectively characterize levels of a participant’s environmental exposure prior to onset of cancer, a feat that can’t be accurately accomplished retrospectively.

Recruiting began in fall 2002 for a pilot phase in four cities—Phoenix, St. Louis, Tampa, and Providence—involving a total of 2,000 participants. “We wanted to go slowly at first,” explains Sandler. The pilot phase gave the researchers time to fine-tune recruiting strategies and data collection methods, arrange for field staff training, and streamline the lengthy questionnaire. The original four cities were chosen for their economic, ethnic, and geographic diversity, Sandler says, and by early 2004 the study spread beyond city limits to encompass the entire states of Arizona, Missouri, Florida, and Rhode Island. In August the study also began recruiting in Illinois, Ohio, Virginia, and North Carolina.

Getting adequate participant diversity is important, Sandler says. She believes the study results will be useful to all U.S. women only if a diverse cross-section of women participate. “With a diverse population, we will have a wider range of [health and environmental] exposures, increasing our ability to detect associations,” she says.

The original eight states were chosen in part because they had community- and church-based breast cancer awareness programs, heavy minority interest, and what Sandler pragmatically terms “good connections” in both public and private sectors. “Our contacts in the breast cancer advocacy community help with grassroots recruitment,” she says.

Participants receive a welcome kit by mail with instructions on how to prepare for the study. A staff member calls first to walk the participant through the kit and later to conduct the survey, which takes about two hours. Next, independent phlebotomists working under contract to the NIEHS make home visits to draw blood samples, collect household dust samples and toenail clippings, and take blood pressure, weight, height, and body measurements.

“Even though it’s an enormous national study, we’re doing everything we can to make it as personal as possible,” Sandler says. “We want to make sure that the women get something back, thus we have a duty to let them know what we learn from the study. We plan to contact them regularly over the years with news from the study.”

Sandler and Weinberg will closely evaluate the expected 1,500 women who will develop breast cancer within five years of the study’s start, analyzing environmental, genetic, and health data captured from the very beginning. “We’ve learned what works [in terms of study design and implementation], and what doesn’t,” Sandler says. “We’re ready.”

## Figures and Tables

**Figure:**
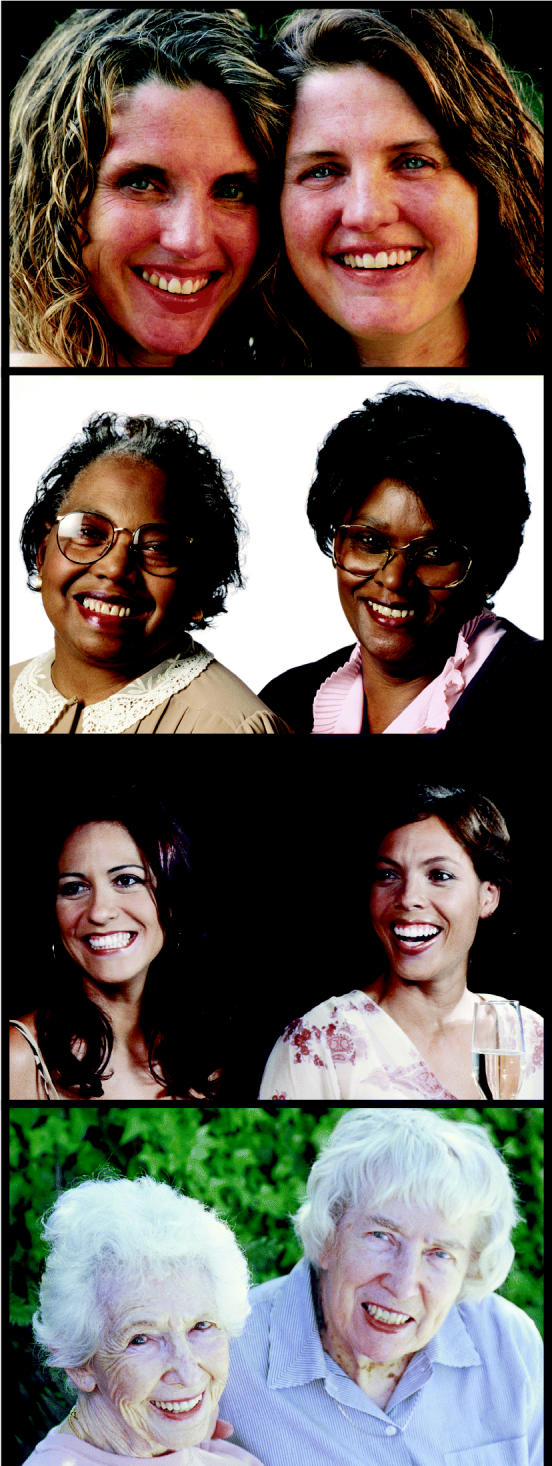
**Sisters are doing it for themselves.** The Sister Study, now recruiting nationwide, will yield new information on how genes and environment may interact to cause breast cancer.

